# Genomic data-sharing: what will be our legacy?

**DOI:** 10.3389/fgene.2014.00034

**Published:** 2014-03-05

**Authors:** Shawneequa Callier, Rajah Husain, Rachel Simpson

**Affiliations:** ^1^Department of Clinical Research and Leadership, School of Medicine and Health Sciences, The George Washington UniversityWashington, DC, USA; ^2^School of Public Health and Health Services, The George Washington UniversityWashington, DC, USA

**Keywords:** privacy, ELSI, genetic research, personalized medicine, ethics, medical

## Abstract

Prior to 1974, the Tuskegee Syphilis experiments, expansive use of the HeLa cells, and other blatant instances of research abuse pervaded the medical research field. Ongoing challenges to informed consent, privacy and data-sharing will influence the stories that research participants today share with future generations. This has significant implications for the advancement of genomic science, and the public's perception of genomic research.

## Introduction

Personalized genomic medicine (PGM)—medical care that is tailored to individuals based on their genetic makeup—has the potential to revolutionize the way providers diagnose and treat patients. By testing patients for clinically validated pharmacogenomic biomarkers, for instance, physicians will be able to tailor drug selection, dosage levels, and treatment duration in ways that avoid adverse drug reactions (Ramos et al., 2013). In many clinics, physicians are already utilizing this technology.

Various bottlenecks and rate-limiting research policies, however, are preventing medical providers and patients from realizing the full potential of PGM (Ramos and Rotimi, 2009; Ramos et al., 2012). Insufficient enrollment of ethnically diverse populations in genomic research and the use of vague racial and ethnic categories to describe research participants are examples of widespread practices that have led to knowledge gaps in PGM (Lee, 2009; Rotimi and Jorde, 2010; Ramos et al., 2013). This has extensive implications for scientists as well as patients, because knowledge of the penetrance and significance of key genetic markers will remain limited until large genomic datasets from diverse populations are developed and explored for the benefit of patients globally (Ramos et al., 2012, 2013)—causing frustration among investigators as well as clinicians who struggle to understand the clinical utility of certain genetic tests in diverse populations.

Fortunately, scientists see the urgency for reform, and have the advanced digital tools and sequencing technologies to develop and analyze datasets that are more representative of the global population. Further, because of the scientific value of large genomic data-sets, prevailing regulations and policies favor a collaborative research environment that encourages data-sharing across international jurisdictions. These advances are not without consequence, of course, and the future impact of the unprecedented data-sharing and storing that is set to take place in the coming years intensifies longstanding challenges related to informational privacy and public expectations about benefit sharing and control over biomedical samples.

A recent NIH data-agreement with the family of Henrietta Lacks, whose whole genome sequence was published on the world-wide-web without her relatives' consent and then removed in response to staunch public disapproval, provides an example of how innovation and communication can help bridge the cultural, legal, and ethical divides between researchers located in one environment and the original owners of the samples and data located in different states, countries, and regions.

As we move forward into the future and into the era of “big-data,” what protocols and standards will prevent this type of breach from happening again? Who will be held accountable if an unauthorized data breach undermines the narratives, identities, and values of individuals whose DNA sequences become available for public knowledge or scrutiny? How can investigators and research participants ensure that samples donated to research and perhaps, governed strictly locally, do not become vulnerable once they leave local jurisdictions?

## Lessons from HeLa

In 1950, Henrietta Lacks, an African-American woman, wife and mother of five children, entered the public ward of Johns Hopkins University complaining of a “knot on her womb” (Skloot, 2010). Soon after, she was diagnosed with an aggressive form of cervical cancer that took her life less than a year later. Unbeknownst to Lacks, however, a small part of her cancer tumor lived on. As was common practice at the time, Lacks' treating physician collected her human cell tissue without her permission or knowledge, labeled it “HeLa” using the initials of her first and last name, and stored her specimens in a laboratory where he was endeavoring unsuccessfully to grow an immortal line of cancer cells. For the first time in the laboratory (and possibly in history), these cancer cells multiplied and survived, creating a durable line of cell culture that the laboratory went on to share with anyone who asked for them (Skloot, 2010).

Twenty-four years later, in 1974, the United States Congress passed the National Research Act (NRA), which led to the publication of the Belmont Report (a foundational document in biomedical research ethics) and the development of legal protections for human research participants engaging in federally funded research (The National Commission for the Protection of Human Subjects of Research, 1979; The Office for Human Research Protection, 1991). The Common Rule requires that researchers obtain informed consent to use the identifiable research samples and data of patients and research participants, or else that they de-identify the samples before sharing them. The Belmont report requires that investigators respect participants in research and treat them in an ethical manner by honoring their autonomous decisions, protecting them from harm, and treating them justly and fairly. Today, it is clear that investigators operating during Lacks' life time lacked clear guidance on these principles. Members of the public and research community, for instance, have pointed to disrespect for Lacks' private life and autonomy, and the unjust enrichment of investigators who have benefited from their research on the HeLa cells while her family remained poor (Javitt, 2010; Skloot, 2010). While various scholars in the humanities disciplines, including those funded by the ethical, legal, and social issues (ELSI) in genetics program, have worked diligently to identify and understand how to address ethical issues raised by genomic research, including those values at the heart of the controversy around the use of the HeLa cells, there remains much debate about how to best protect the privacy of individuals who participate in genomic research.

Recent publication of Henrietta Lacks' genome sequence on the internet provides a case in point. Although the HeLa story has caused a stir across the United States—leading to a public apology by Johns Hopkins University to the Lacks family—Skloot's publication did not dissuade researchers in Germany from publishing her full genome earlier this year in 2013, again without prior consent (Skloot, 2013). Researchers and journal editors, while still following all rules, had seemingly overlooked any questions about privacy and consent for the HeLa genome (National Institutes of Health Advisory Committee to the Director HeLa Genome Data Access Working Group, 2013). The public, however, was not forgiving. The public posting evoked visceral reactions among researchers, patient advocates, bioethicists and members of Lacks' family who were concerned about the privacy implications for Lacks and her descendants (National Institutes of Health Advisory Committee to the Director HeLa Genome Data Access Working Group, 2013). In response, the investigators removed Lack's genomic sequence from the public domain and other papers were put on hold while the National Institutes of Health (NIH) initiated a negotiation with the Lacks family (National Institutes of Health Advisory Committee to the Director HeLa Genome Data Access Working Group, 2013).

After a series of talks, NIH established the HeLa Genome Data Access Working Group of the Advisory Committee to the Director, which is a special committee charged with reviewing the release of data from the HeLa Genome with input from two serving members of the Lacks family (National Institutes of Health Advisory Committee to the Director HeLa Genome Data Access Working Group, 2013). These laudable efforts on the part of leadership in the biomedical research community demonstrate a swift and thoughtful response to the Lacks family's disgruntlement with the investigators' failure to consult with them about publicizing information that could reveal heritable traits and information about Lacks and her descendants. They also demonstrate the potential for public backlash against policies perceived to be contrary to accepted ethical norms.

## What legacy will we leave for research participants of this generation?

The expectations for researchers are rapidly changing. Consensus is building among stakeholders, for instance, that research results that can benefit participants and their families should be returned to them, because of the important health and reproductive predispositions that genomic information may reveal (Henderson et al., 2012). Some scholars have argued that there will be greater demand among research participants who believe they have a right to access the information in their genomes (Henderson et al., 2008; McGuire et al., 2011). Research has also shown that members of the public have privacy preferences that are out of tune with long standing privacy policies applied to research (McGuire et al., 2008, 2011; McGuire and Beskow, 2010; McGuire and Lupski, 2010). Increasingly, participants are favoring more restrictive data release/sharing options, and at higher rates—in one study, nearly half (47%: McGuire et al., 2011) of the participants selected to release their data on a restricted basis.

In addition, the general public is knowledgeable and concerned about how genomic test results may affect future medical treatment or insurance coverage. Although it is prohibited under the Genetic Information Nondiscrimination Act for health insurance groups to use genetic information to determine eligibility and coverage, the confidentiality of such information remains a concern for individuals, especially those living in underserved and ethnically diverse communities (Goldenberg et al., 2013).

As exhibited by the research community's response to the public reaction to publication of the HeLa genome, privacy and consent issues are likely to become predominant concerns in the public psyche. An author at Slate Magazine, for instance, outlined the questions that are likely on the minds of the readers of this popular news source, “… the prevailing theme of Skloot's book is not on the question of how much Lacks and her family are owed; rather, it's that Lacks' doctors experimented on and distributed her cells without asking, or telling, her or her family” (Singer-Vine, 2010). Meanwhile a writer for Essence magazine explained, “[t]he big thing with Henrietta's cells is the privacy violation. Her medical records were published at one point by a journalist so there are these questions about her privacy. There are also a lot of questions about whether you should take something from somebody without asking” (Watts, 2012). As other countries build their own genomic research laboratories and data collection processes, it is becoming clear that the research community has an opportunity here to build a legacy that will influence the public's opinion on research, data-sharing and informational privacy for years to come.

Currently, a solution that receives great support involves including language in the consent form warning potential research subjects that they will lose control and ownership over their samples and data, and that reidentification is possible. In addition, there are movements to educate the public about the benefits of genomic research (Green et al., 2011). These mechanisms, however, will not protect the family members of research participants who are indirectly linked with research results, prevent the stigmatization of communities and the community members whose DNA samples were collected in one jurisdiction and insufficiently secured in another, or adequately buffer the damaging impact that high profile privacy breaches may have on research. Community engagement helps to educate potential research subjects in a culturally competent manner about the risks and benefits of participation in genetic research, but to be truly effective, sound mechanisms that support fair negotiation among researchers from different nations and that hold violators accountable must be in place.

At the time of this publication, it will have been 81 years since the Tuskegee Syphilis Study began in Tuskegee, Alabama and 41 years since it was terminated. Although egregious physical, mental, and emotional research abuses like those that occurred at the hands of the Public Health Service (PHS) have been outlawed, many individuals remain skeptical of research. The Tuskegee Syphilis Study, in particular, is often referenced as a reason for why self-identified African-Americans are wary of participating in biomedical research. The legacy of the PHS study remains indelible in the minds of many as one of the most infamous biomedical research studies. What will research participants remember about their participation in genetic research 50 years from today?

### Conflict of interest statement

The authors declare that the research was conducted in the absence of any commercial or financial relationships that could be construed as a potential conflict of interest.
